# Spontaneous Life-Threatening Hemorrhage Secondary to a Peritonsillar Abscess Requiring Intervention and Local Flap Reconstruction

**DOI:** 10.7759/cureus.105661

**Published:** 2026-03-22

**Authors:** Alejandro Santiago-Nazario, Carlos Crespo-Borges, Edgar Del Toro-Diez, Giovanny Pérez, Shayanne A Lajud

**Affiliations:** 1 Otolaryngology - Head and Neck Surgery, University of Puerto Rico School of Medicine, San Juan, PRI

**Keywords:** buccal fat pad flap, oropharyngeal reconstruction, peritonsillar abscess, quinsy tonsillectomy, spontaneous tonsillar hemorrhage

## Abstract

Spontaneous tonsillar hemorrhage (STH) is a rare but potentially life-threatening complication most commonly associated with tonsillitis and, less frequently, peritonsillar abscess. Prompt recognition and management are critical due to the risk of airway compromise and vascular injury. We report the case of a 30-year-old female who developed massive oropharyngeal hemorrhage following spontaneous drainage of a peritonsillar abscess, requiring emergent airway protection and surgical intervention.

The patient underwent Quinsy tonsillectomy, which revealed an extensive defect created by the hematoma communicating with the parapharyngeal and submandibular spaces, placing major cervical vessels at risk. Given the size and depth of the defect, reconstruction was performed using a pedicled buccal fat pad flap to provide vascularized coverage of exposed deep neck spaces. Computed tomography angiography demonstrated close proximity of the peritonsillar space to the facial artery without evidence of pseudoaneurysm or active extravasation. The patient had an uneventful recovery with complete mucosalization of the reconstructed oropharyngeal defect.

This case highlights that spontaneous tonsillar hemorrhage following peritonsillar abscess drainage may result in extensive deep neck space involvement requiring not only definitive tonsillectomy but also reconstructive intervention. The successful use of a buccal fat pad flap in this non-oncologic infectious setting expands reconstructive options for complex oropharyngeal defects and underscores its utility in preventing secondary hemorrhage and vascular complications.

## Introduction

Spontaneous tonsillar hemorrhage (STH) is a rare and potentially life-threatening complication, typically arising in the context of acute or chronic tonsillitis. STH is defined as bleeding from the tonsil without an identifiable iatrogenic cause and is characterized by continuous bleeding lasting more than one hour or blood loss exceeding 250 mL [1]. Although the overall incidence is relatively low at around 1.2%, tonsillitis remains the most frequent predisposing factor, accounting for over 80% of STH cases [1,2].

An understanding of the regional anatomy is essential to appreciate the potential severity of this condition. The palatine tonsils reside within the tonsillar fossa, bounded laterally by the superior pharyngeal constrictor muscle. Immediately deep to this muscle lies the peritonsillar space, a potential space composed of loose areolar tissue. Critically, this region is in close proximity to major cervical vascular structures, including branches of the external carotid artery, such as the tonsillar branch of the facial artery, ascending palatine artery, and dorsal lingual branches, as well as the internal carotid artery, which courses posterolaterally to the tonsillar fossa. Venous drainage via the peritonsillar plexus further contributes to the vascular density of this area.

Peritonsillar abscess formation can lead to progressive inflammation, tissue necrosis, and increased local pressure within this confined space. When spontaneous drainage occurs, the rupture of inflamed and friable tissue may expose or erode adjacent arterial or venous structures. Additionally, enzymatic degradation and septic thrombophlebitis may weaken vessel walls, predisposing them to sudden hemorrhage. Thus, spontaneous decompression of a peritonsillar abscess, while occasionally relieving pressure-related symptoms, can paradoxically precipitate significant bleeding if adjacent vascular structures are compromised.

Historically, prior to the advent of antibiotics, major vessel erosion due to deep neck abscesses was a relatively common cause of fatal hemorrhage [3]; however, such severe cases have become exceedingly rare in contemporary practice.

We present the case of a 30-year-old female patient who experienced profuse STH following spontaneous drainage of a peritonsillar abscess, which necessitated acute airway management and surgical intervention.

## Case presentation

**Figure 1:**
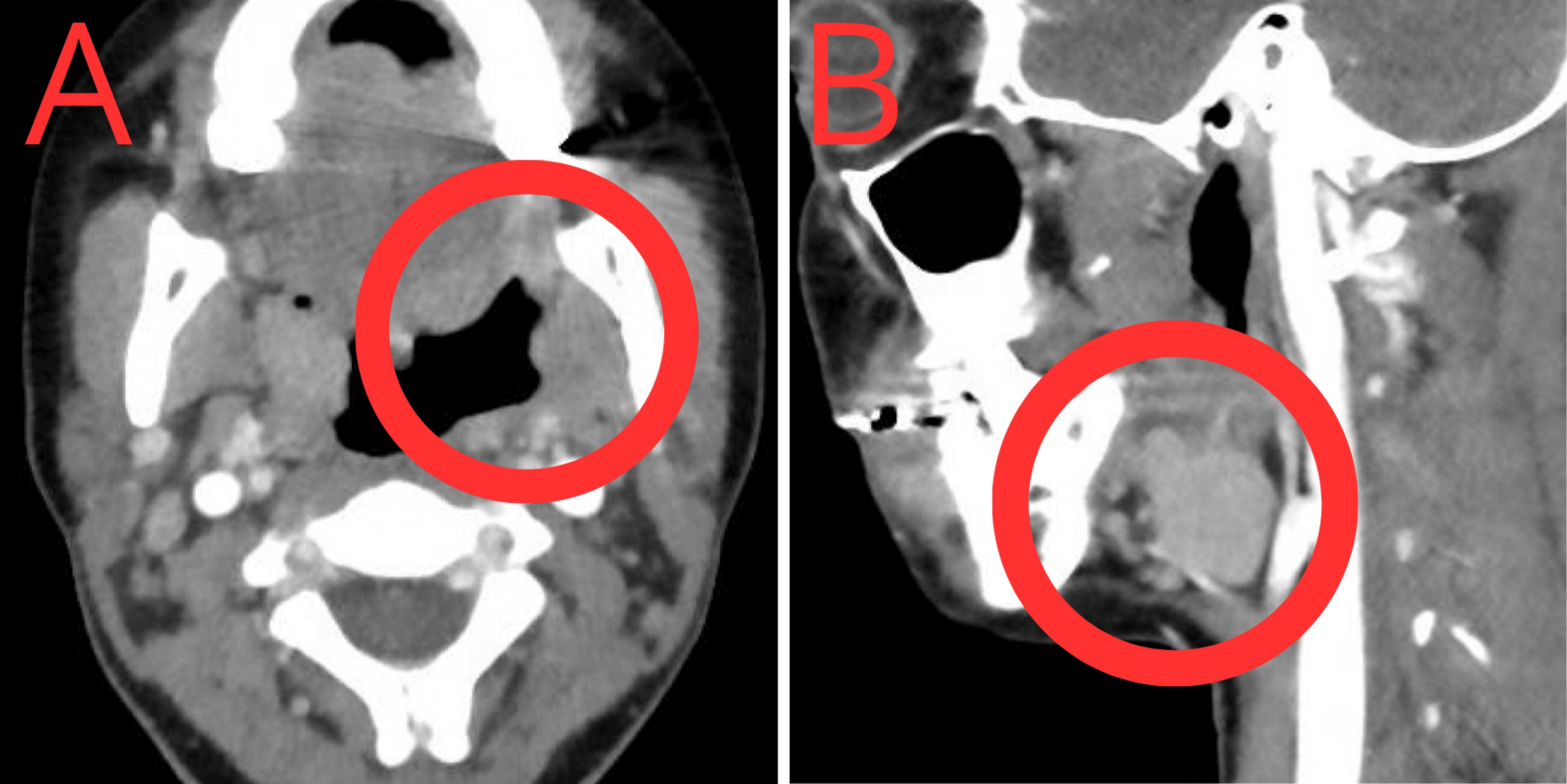
Axial (A) and sagittal (B) views demonstrate a left peritonsillar fluid collection with surrounding inflammatory changes (red circles), consistent with a peritonsillar abscess prior to spontaneous drainage

**Figure 2:**
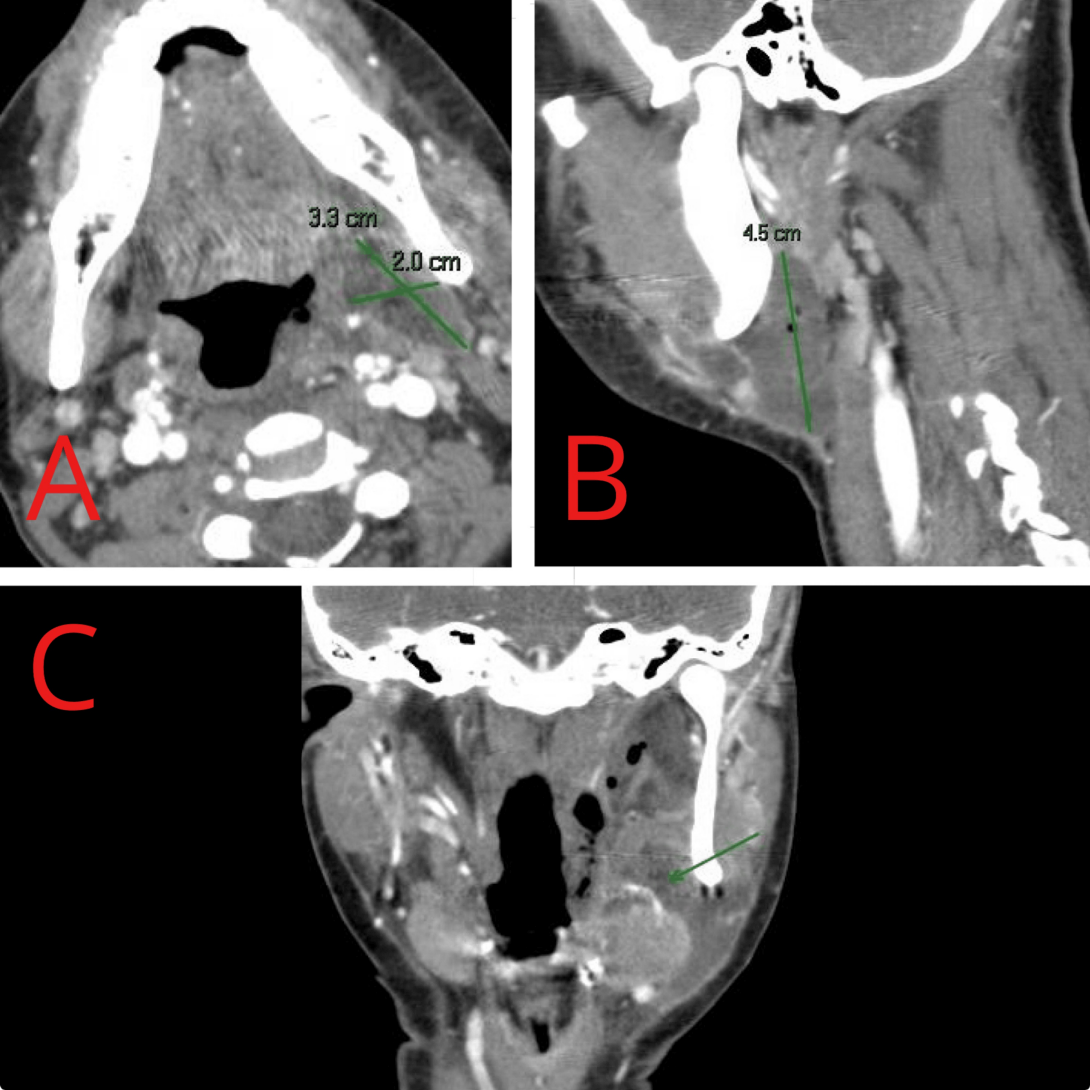
Axial (A) and sagittal (B) views demonstrate a residual rim-enhancing fluid collection with internal gas locules centered within the left parapharyngeal and masticator spaces. Coronal (C) view highlights the close anatomical relationship between the residual collection and a segment of the left facial artery (arrow), without evidence of active contrast extravasation or pseudoaneurysm formation.

An otherwise healthy, 30-year-old female presented with a peritonsillar abscess to an outside hospital (Figure 1). Peritonsillar abscesses classically present with severe unilateral throat pain, trismus, muffled “hot potato” voice, and a bulging tonsil with uvular deviation; in rare cases, spontaneous rupture may be followed by sudden oropharyngeal bleeding. In this patient, the abscess was managed with intravenous antibiotics, and the following day, it was noted to be draining spontaneously. That same day, she developed profuse oropharyngeal bleeding, which led to airway compromise, requiring emergent endotracheal intubation and throat packing. The patient was transferred to our institution for definitive otolaryngologic surgical management. A CT angiogram revealed a close anatomical relationship between the left peritonsillar space and a short segment of the left facial artery, while ruling out pseudoaneurysms or active extravasation (Figure 2).

In the operating room, oropharyngeal exposure revealed that the left tonsil had detached from the superior constrictor muscle and the superior tonsillar pole, leaving it attached only to the inferior tonsillar pole. A Quinsy tonsillectomy was completed. Upon removal of the tonsil and exploration of the tonsillar fossa, parapharyngeal fat and vessels were identified. Intraoperatively, the bleeding vessel was identified. A defect measuring approximately 2 cm wide × 6 cm long was observed, with a noticeable inferior tract extending into the ipsilateral visceral and submandibular spaces. Given the potential exposure of the great vessels of the neck at the level of the parapharyngeal space in the setting of a deep neck space abscess, the decision was made to reconstruct the defect using a left buccal fat pad. A horizontal incision was performed beneath Stensen’s duct papilla in the buccal mucosa. The buccinator muscle was incised, and the buccal fat pad was mobilized with blunt dissection. It was left attached at its vascular pedicle in the cheek and advanced into the defect in the ipsilateral oropharynx. The fat pad was secured in place with 2-0 Vicryl sutures, and Tisseel (Baxter, Deerfield, Illinois, US) was used to reinforce the reconstruction.

The patient was subsequently extubated and transferred to the intensive care unit (ICU). She was observed on intravenous antibiotics for an additional three days and was later discharged home on oral antibiotics. At follow-up three weeks later, examination revealed a well-mucosalized and fully healed oropharyngeal defect.

## Discussion

STH is an uncommon but potentially life-threatening complication of acute tonsillitis and peritonsillar abscess. It is classically defined as continuous tonsillar bleeding lasting more than 1 hour or involving blood loss greater than 250 mL [1]. Although the reported incidence is low, tonsillitis accounts for over 80% of documented cases, with peritonsillar abscess representing a higher-risk subset due to proximity to major cervical vessels [2].

Historically, prior to the widespread use of antibiotics, hemorrhage from peritonsillar and parapharyngeal infections was frequently fatal, often requiring carotid artery ligation to achieve hemostasis [3]. While such outcomes are now rare, contemporary reports continue to describe severe hemorrhage associated with vessel erosion following abscess formation or spontaneous drainage, even in patients receiving appropriate antimicrobial therapy [2,4].

The pathophysiology of STH is believed to involve inflammatory erosion of peripheral tonsillar vessels rather than true aneurysm formation [1]. In the present case, computed tomography angiography demonstrated a close anatomical relationship between the left peritonsillar space and the facial artery without evidence of pseudoaneurysm, supporting inflammation-mediated vessel compromise as the most likely etiology [5].

Management strategies depend on the severity of hemorrhage. Conservative measures, including topical vasoconstrictors, cauterization, or packing, may be effective in mild cases; however, persistent or massive bleeding necessitates airway protection and definitive surgical intervention [2,6]. Quinsy tonsillectomy provides direct access for hemostasis, removal of infected tissue, and prevention of recurrent hemorrhage, and remains the treatment of choice in life-threatening presentations.

What distinguishes this case is the extent of the residual defect following evacuation of the hematoma and infected tissue, with direct communication into the parapharyngeal and submandibular spaces, placing the carotid artery and its branches at theoretical risk. While post-tonsillectomy hemorrhage occurs in up to 5.4% of cases, life-threatening bleeding is reported in approximately 1%, based on a large transoral oropharyngectomy series, and typically does not involve exposed deep neck spaces [7].

Reconstruction of extensive oropharyngeal defects is more commonly discussed in oncologic surgery. Local and regional flaps, including buccinator myomucosal and buccal fat pad (BFP) flaps, have demonstrated reliable outcomes for lateral oropharyngeal wall reconstruction [8,9]. The BFP flap offers several advantages, including ease of harvest, robust vascularity, minimal donor-site morbidity, and rapid mucosalization [9-11]. Although its use is well-established in reconstructive and oncologic settings, its application in complex infectious defects of the oropharynx is less frequently described.

A focused literature search of PubMed and Google Scholar was performed using the keywords “buccal fat pad flaps,” “spontaneous tonsillar hemorrhage,” “spontaneous tonsillar bleeding,” “quinsy tonsillectomy,” “tonsillar bleeding,” and “peritonsillar abscess,” without date restriction, and revealed limited documentation of buccal fat pad reconstruction in the setting of spontaneous tonsillar hemorrhage following peritonsillar abscess.

In this case, a pedicled buccal fat pad flap provided effective coverage of exposed deep neck spaces adjacent to major cervical vessels, with the goal of reducing the risk of secondary hemorrhage and persistent infection. Rather than representing a novel technique per se, this approach illustrates a pragmatic and adaptable use of established reconstructive principles in a high-acuity infectious scenario. In centers accustomed to managing complex head and neck pathology with limited resources, thoughtful application of regional flaps, such as the BFP, can expand surgical options when confronted with extensive defects and limited local tissue coverage.

## Conclusions

Although rare, spontaneous tonsillar hemorrhage should be considered a potentially life-threatening complication of peritonsillar abscesses. This case highlights both the importance of prompt airway control and definitive surgical management, as well as the potential for extensive deep neck space involvement following abscess drainage. The successful use of a pedicled buccal fat pad flap in this setting demonstrates its versatility as a reconstructive option for extensive non-oncologic oropharyngeal defects and may broaden surgical strategies for managing similarly complex infectious cases.

## References

[1] Griffies WS, Wotowic PW, Wildes TO (1988). Spontaneous tonsillar hemorrhage. Laryngoscope.

[2] Salem A, Healy S, Pau H (2010). Management of spontaneous tonsillar bleeding: review. J Laryngol Otol.

[3] Salinger S, Pearlman SJ (1933). Hemorrhage from pharyngeal and peritonsillar abscesses. Report of cases, review of the literature and discussion of ligation of the carotid artery. Arch Otolaryngol.

[4] Kim YS, Hong SJ, Choi J, Lee SH, Kwon SY, Choi JH (2010). Spontaneous tonsillar hemorrhage and post-tonsillectomy hemorrhage. Clin Exp Otorhinolaryngol.

[5] Nguyen DV, Tran DH, Champ KG, Vutukuri S, Verceles A (2022). Spontaneous oropharyngeal hemorrhage complicated by cirrhosis, resulting in hemorrhagic shock. Am J Case Rep.

[6] Johnson RF, Stewart MG (2005). The contemporary approach to diagnosis and management of peritonsillar abscess. Curr Opin Otolaryngol Head Neck Surg.

[7] Pollei TR, Hinni ML, Moore EJ, Hayden RE, Olsen KD, Casler JD, Walter LC (2013). Analysis of postoperative bleeding and risk factors in transoral surgery of the oropharynx. JAMA Otolaryngol Head Neck Surg.

[8] Jung BK, Song SY, Kim SH (2015). Lateral oropharyngeal wall coverage with buccinator myomucosal and buccal fat pad flaps. Arch Plast Surg.

[9] Rapidis AD, Alexandridis CA, Eleftheriadis E, Angelopoulos AP (2000). The use of the buccal fat pad for reconstruction of oral defects: review of the literature and report of 15 cases. J Oral Maxillofac Surg.

[10] Dean A, Alamillos F, García-López A, Sánchez J, Peñalba M (2001). The buccal fat pad flap in oral reconstruction. Head Neck.

[11] Egyedi P (1977). Utilization of the buccal fat pad for closure of oro-antral and/or oro-nasal communications. J Maxillofac Surg.

